# The Low Temperature Induced Physiological Responses of *Avena nuda* L., a Cold-Tolerant Plant Species

**DOI:** 10.1155/2013/658793

**Published:** 2013-06-11

**Authors:** Wenying Liu, Kenming Yu, Tengfei He, Feifei Li, Dongxu Zhang, Jianxia Liu

**Affiliations:** ^1^School of Life Science, Shanxi Datong University, Datong 037009, China; ^2^School of Agriculture and Food Science, Zhejiang Agriculture and Forestry University, Hangzhou 311300, China

## Abstract

The paperaim of the was to study the effect of low temperature stress on *Avena nuda* L. seedlings. Cold stress leads to many changes of physiological indices, such as membrane permeability, free proline content, malondialdehyde (MDA) content, and chlorophyll content. Cold stress also leads to changes of some protected enzymes such as peroxidase (POD), superoxide dismutase (SOD), and catalase (CAT). We have measured and compared these indices of seedling leaves under low temperature and normal temperature. The proline and MDA contents were increased compared with control; the chlorophyll content gradually decreased with the prolongation of low temperature stress. The activities of SOD, POD, and CAT were increased under low temperature. The study was designated to explore the physiological mechanism of cold tolerance in naked oats for the first time and also provided theoretical basis for cultivation and antibiotic breeding in *Avena nuda* L.

## 1. Introduction 

*Avena nuda* L., also named naked oats, is originated and widely separated in north and high altitude region in China. It belongs to herb of gramineous plant with annual growing. Naked oats has great value in nutrition and medicine. It contains abundant proteins with 18 kinds of amino acid, and lots of unsaturated fatty acids. Naked oats likes to grow under cool weather and has tolerance to low temperature. Naked oats is a typical temperate crop adapted to cool climates. 

Low temperature is a major abiotic stress that limits the growth, productivity, and geographical distribution of agricultural crops and can lead to significant crop loss [1, 2]. To cope with low temperature, plants have evolved a variety of efficient mechanisms that allow them to adapt to the adverse conditions [3, 4]. This adaptive process involves a number of biochemical and physiological changes, including increased levels of proline, soluble sugars, and MDA, as well as enzyme activities [5].

Understanding the mechanisms of low temperature adaptation is crucial to the development of cold-tolerant crops. The study was designated to explore the physiological mechanism of cold tolerance in naked oats. The responses of the *Avena nuda* L. seedlings to low temperature stress were also evaluated by measuring electrolyte leakage (EL), chlorophyll content, and the concentration of MDA. We measured and compared these indices of seedlings leaves under low temperature and normal temperature. The study provided theoretical basis for cultivator and antibiotic breeding in *Avena nuda* L.

## 2. Materials and Methods

### 2.1. Materials and Cold Treatment

Naked oats cultivar Jinyan 14 (*Avena nuda* L.) was used in the experiment. Seeds were sterilized by incubation for 1 min in 75% ethanol and then washed thoroughly with sterile water. The seeds were germinated in soil in pots at 20°C under long-day conditions (16 h of cool white fluorescent light, photon flux of 70 umol m^−2^ s^−1^). 

Seedlings at the four-leaf stage were subjected to cold stress. Plants were divided into three groups, one group was under normal temperature as control, the other two were subjected to low temperature processing, and each group had the stress repeated three times. Seedlings of the control group were grown at 20°C continuously. For low temperature treatments, seedlings were transferred to a temperature of 1°C and −10°C in an artificial climate box under the same light and photoperiodic conditions for 7 days. 

The leaves were sampled after 0, 1, 3, 5 and, 7 d of treatment for next measurement. The leaf samples were immediately frozen in liquid nitrogen and stored at −80°C until use. Three independent biological samples for each treatment were harvested, and each replicate contained 10 plants.

### 2.2. Determination of Relative Electrolyte Leakage

For electrolyte leakage measurement, protocol was used as described [6]. Briefly, 100 mg leaves were placed in 25 mL distilled water, shaken on a gyratory shaker (200 rpm) at room temperature for 2 h, and the initial conductivity (*C*1) was measured with a conductivity instrument. The samples were then boiled for 10 min to induce maximum leakage. After cooling down at room temperature, electrolyte conductivity (*C*2) was measured, and the relative electrical conductivity (*C*%) was calculated based on (*C*1/*C*2) × 100. All low temperature testing experiments were repeated three times. A paired *t*-test was used to determine the difference between the cold treatment and normal condition.

### 2.3. Determination of Chlorophyll Content

For estimation of total chlorophyll, protocol was followed as described [7]. About 100 mg of fine powder of leaf tissue was homogenized in 1 mL of 80% acetone and kept for 15 min at room temperature in dark. The crude extract was centrifuged for 20 min at 10,000 rpm at room temperature, and the resultant supernatant was used for assessing absorbance at 633 and 645 nm with a spectrophotometer. Total chlorophyll content was computed in terms of fresh weight (FW).

### 2.4. Determination of Proline Content

Proline concentrations in naked oats leaves were measured by the sulfosalicylic acid-acid ninhydrin method with slight modifications [8]. Around 100 mg of tissues were used and extracted in 5 mL of 3% sulphosalicylic acid at 95°C for 15 min. After filtration, 2 mL of supernatant was transferred to a new tube containing 2 mL of acetic acid and 2 mL of acidified ninhydrin reagent. After 30 min of incubation at 95°C, samples were kept at room temperature for 30 min and 5 mL of toluene was added to the tube with shaking at 150 rpm to extract red products. The absorbance of the toluene layer was determined at 532 nm using spectrophotometer.

### 2.5. Determination of Malondialdehyde (MDA) Content

Malondialdehyde (MDA) content in naked oats leaves was determined following the protocols as described [9]. Briefly, leaves were homogenized in 5 mL of 10% trichloroacetic acid containing 0.25% thiobarbituric acid. The mixture was incubated in water at 95°C for 30 min, and the reaction was stopped in an ice bath. The mixture was centrifuged at 10,000 g for 20 min, and the absorbance of the supernatant was measured at 450, 532, and 600 nm. 

### 2.6. Determination of Peroxidase, Superoxide Dismutase, and Catalase Activity

Naked oats leaves (0.5 g) were ground thoroughly with a cold mortar and pestle in 50 mmol potassium phosphate buffer (pH 7.8) containing 1% polyvinylpyrrolidone. The homogenate was centrifuged at 15,000 g for 20 min at 4°C. The supernatant was crude enzyme extraction. The activities of peroxidase (POD), superoxide dismutase (SOD), and catalase (CAT) were measured using the protocols described [10]. 

## 3. Results and Discussion

### 3.1. Phenotypic Changes under Cold Stress

**Figure 1:**
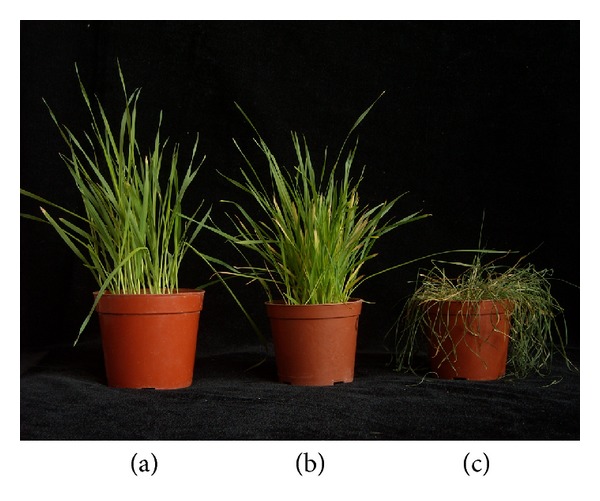
The seedlings of naked oats at normal temperature (a) and low temperature of 1°C (b) and −10°C (c) after 7 days.

We investigated the phenotypic response to low temperature. During the cold treatment of 1°C, naked oats grew well as usual. Until 5 days later, the seedlings were always strong only except some leaf apexes began to get yellow. In the 7th day, most parts of seedling remained green as normal temperature as shown in Figure 1. The seedling got curl after 3-4 hours after exposure to −10°C cold stress. Some leaf began to get yellow and curled seriously in the third day, while the seedling grew slowly. Most leaves showed severe rolling and wilting in the 7th day.

No obvious differences have been found in growth and developed between normal temperature and at 1°C, indicating that the naked oats have cold tolerance at the low temperature of 1°C.

### 3.2. Changes of Relative Electrolyte Leakage under Cold Stress

**Figure 2:**
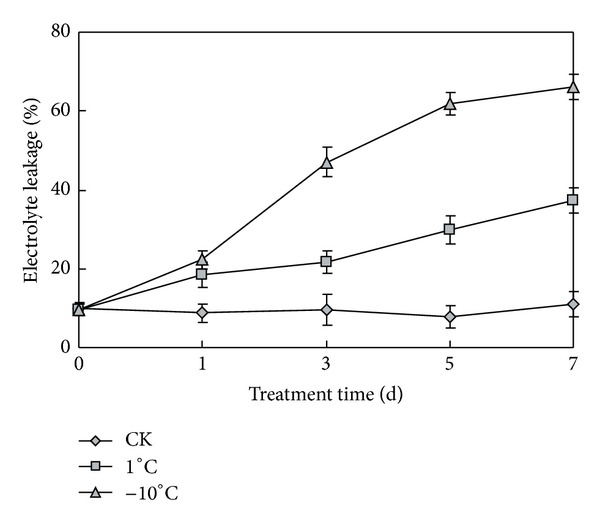
Relative electrolyte leakage of seedling leaves under normal and low temperatures. CK represents normal growth condition. Each value is the average ± standard error (±SE) result of three independent measurements.

Cold stress often causes damage to cell membranes, so, we tested the cell-membrane penetrability. Cell membrane penetrability was evaluated by the relative conductance of the cell membrane under cold stress [11, 12]. The electrolyte leakage test was performed to compare membrane integrity [13]. For such experiment, plants were subjected to low temperature of 1°C and −10°C. The relative electrolyte leakage of seedling leaves was increased greatly with cold treatment as shown in Figure 2. There was no significant difference under normal temperature; the average electrolyte leakage was about 9.7%. During the cold stress, the relative electrolyte leakage value gradually increased with the prolongation of low temperature stress. The electrolyte leakage of −10°C was significantly higher than that of 1°C growing plants. In the 7th day, elative electrolyte leakage increased to 66.0% under cold stress of −10°C, while 37.3% at 1°C and the normal condition still 11.0%. 

When the plants were under low temperature stress, the structure of cellular membrane was damaged. The degree of cell membrane injury induced by cold stress can be reflected by intracellular electrolyte leakage rate. The relative conductance value is one of the effective indicators to indirectly evaluate plant response ability to low temperature stress [14]. The damage degree of cellular membrane was aggravated with the continuity of low temperature stress [12]. In the experiment, the electrolyte leakage in leaves of naked oats seedlings gradually increased under low temperature of 1°C stress, indicating that the damage of low temperature on cell membrane increased gradually. The electrolyte leakage increased more significantly at −10°C than 1°C, indicating that cell membrane was damaged seriously at −10°C than 1°C. At 1°C, the naked oats had some tolerance to protect the cell membrane avoiding cold damage.

### 3.3. Changes of Total Chlorophyll Content under Cold Stress

**Figure 3:**
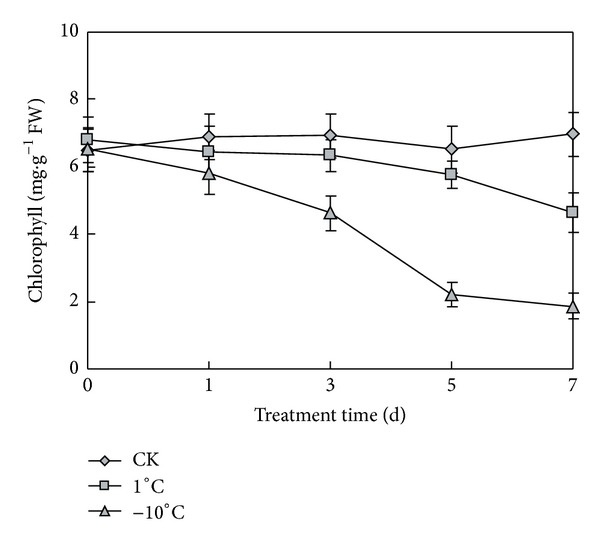
Chlorophyll content of seedling leaves under normal and low temperatures. CK represents normal growth condition. Each value is the average ± standard error (±SE) result of three independent measurements.

Chlorophyll is an extremely important and critical biomolecule in photosynthesis with function of light absorbance and light energy transformation [15]. Low temperature stress can influent plant photosynthesis and decrease the utilization of light [16, 17]. We examined the content of chlorophyll under low temperature of 1°C and −10°C. Compared with the control, the chlorophyll content in seedling leaves under low temperature was lower than that under room temperature. As shown in Figure 3, the change range of chlorophyll content in leaves of naked oats was 6.4–6.9 mg/g under room temperature. At 1°C, the chlorophyll content was decreased with the prolongation of low temperature stress. The chlorophyll decreased slightly from the 1st day to the 5th day, and the content was only slightly less than control, but in 7th day the chlorophyll decreased greatly to 4.6 mg/g, while at −10°C, the chlorophyll content decreased seriously. Especially in the 5th day, chlorophyll content decreased to 2.2 mg/g while 5.8 mg/g at 1°C and 6.5 mg/g at normal temperature. The results indicated that naked oats was damaged less at 1°C than at −10°C. Low temperature inhibits chlorophyll accumulations in actively growing leaves. Naked oats has some degree of cold tolerance at 1°C. 

### 3.4. Changes of Free Proline Content under Cold Stress

Proline is widely distributed in plants as protection material, which is an organic osmolyte [18]. It plays a vital role in maintaining osmotic balance and stabilizing cellular structures in plants. Many plants accumulate free proline in response to abiotic stress of low temperature. Increased free proline content protects the plant against the stress [19].

**Figure 4:**
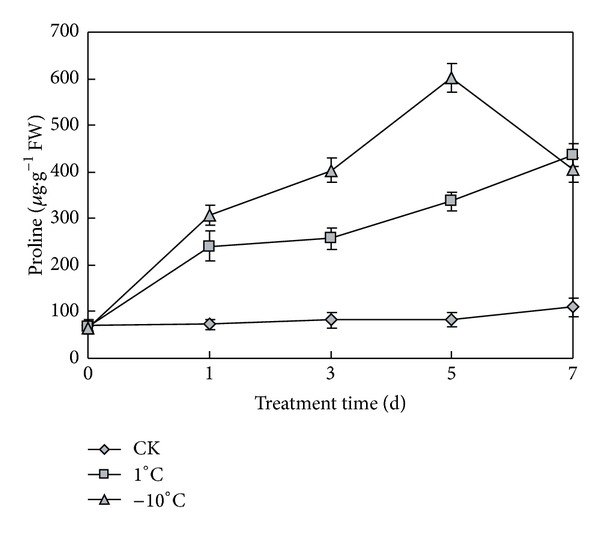
Free proline content of seedling leaves under normal and low temperatures. CK represents normal growth condition. Each value is the average ± standard error (±SE) result of three independent measurements.

The effect of cold on content of proline was investigated. There was no significant difference in proline contents without cold stress as shown in Figure 4. An increase in proline content was observed upon exposure to cold stress. Compared with the control, the free proline content in seedling leaves under low temperature was obviously higher than that under room temperature. At 1°C, the proline content increased with the prolongation of low temperature stress. At −10°C, the proline content was increased in the first five days and reached the max of 601 *μ*g/g in the 5th day, about 6 times of control. Then proline content decreased to 404 *μ*g/g in the 7th day, which was still much higher than control. At −10°C, the plant accumulated more amounts of proline than at 1°C. This indicated that the lower the temperature was, the more proline could be accumulated. 

Proline plays a vital role in maintaining osmotic balance in plants. The accumulation of proline may function in preventing plants from being damaged by stress. The free proline acts as osmolytes to facilitate osmoregulation, thus protecting plants from dehydration resulting from cold stress by reducing water potential of plant cells [20]. In addition, proline can also function as a molecular chaperone to stabilize the structure of proteins as well as play a role in regulation of the antioxidant system [21, 22]. So increased free proline content protects the plant against the stress. The study found greater accumulation of free proline under cold stress, which may partially account for the higher tolerance of plants to cold stress. Accumulation of proline to facilitate osmo-regulation is a common adaptive mechanism for tolerance of plants to abiotic stress. The results were consistent with previous studies that proline accumulated in leaves exposed to cold, salt, and other stresses [23].

### 3.5. Changes of Malondialdehyde (MDA) Content under Cold Stress

**Figure 5:**
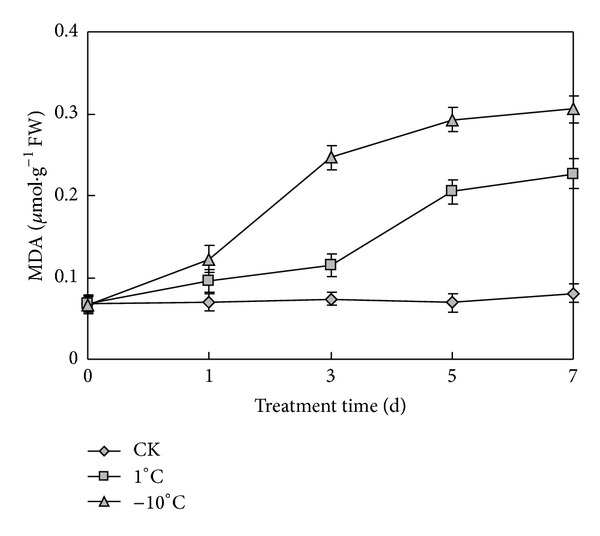
MDA content of seedling leaves under normal and low temperatures. CK represents normal growth condition. Each value is the average ± standard error (±SE) result of three independent measurements.

Cold stress often causes damage to cell membranes. Malondialdehyde (MDA) is an important indicator of membrane system injuries and cellular metabolism deterioration [24]. So, we further measured the cell membrane penetrability. The effects of MDA contents were investigated in the seedling of naked oats. In our experiment, naked oats had a significantly higher MDA level under low temperature stress compared to the control level. As shown in Figure 5, the MDA concentrations increased with the prolongation of low temperature stress. In the seventh day, the MDA content reached to the max nearly 3 times of control at 1°C and about 4 times at −10°C. The increase in MDA content at −10°C was significantly higher than 1°C all the time.

 MDA has been well recognized as a parameter reflecting damage by cold stress. Cell membrane systems are the primary sites of freezing injury in plants [25]. Plants subjected to low temperatures frequently suffer membrane damage, which can be evaluated by relative electrolyte leakage and MDA production. MDA is considered to be the final product of lipid peroxidation in the plant cell membrane [26]. MDA is also an important intermediate in ROS scavenging, and a high level of MDA is toxic to plant cells. In this experiment, in the first day the MDA only increased 0.095 *μ*mol*·*g^−1^ at 1°C, while the MDA concentrations increased to 0.122 *μ*mol*·*g^−1^ at −10°C. In the third day, MDA increased slightly to 0.115 *μ*mol*·*g^−1^ at 1°C, while MDA increased rapidly to 0.247 *μ*mol*·*g^−1^ at −10°C, which was 4 times of control. The increase of MDA at −10°C was greater than at 1°C. These results suggested that cell membrane was little damaged at 1°C at the beginning of cold stress, but was hurt seriously at −10°C. It may have contributed to the different phenotypes under cold stress. Prolonged treatment finally led to a great cell damaged and MDA accumulation. The MDA content increased slowly in first three days at 1°C, but increased rapidly to 0.20 *μ*mol*·*g^−1^ in the fifth day. It indicated that cell membranes of seedlings were injured by cold seriously in 3–5 days at 1°C. 

### 3.6. Changes of Antioxidant Enzymes under Cold Stress

Cold stress induces the accumulation of reactive oxygen species (ROS) such as superoxide, hydrogen peroxide, and hydroxyradicals [27, 28]. The elevated concentrations of ROS can damage cellular structures and macromolecules, leading to cell death [29, 30]. Plants under abiotic stress have evolved a defense system against oxidative stress by increasing the activity of ROS-scavenging enzyme. ROS can be scavenged by superoxide dismutase (SOD), peroxidase (POD), and catalase (CAT) [31]. SOD plays an important role in eliminating ROS induced by cold [32]. POD and CAT can scavenge H_2_O_2_. SOD, POD, and CAT are important enzymes protecting membrane system. Many kinds of antioxidant enzyme activities have been changed under low stress. Changes of enzyme activities are relvant to cold tolerance. 

**Figure 6:**
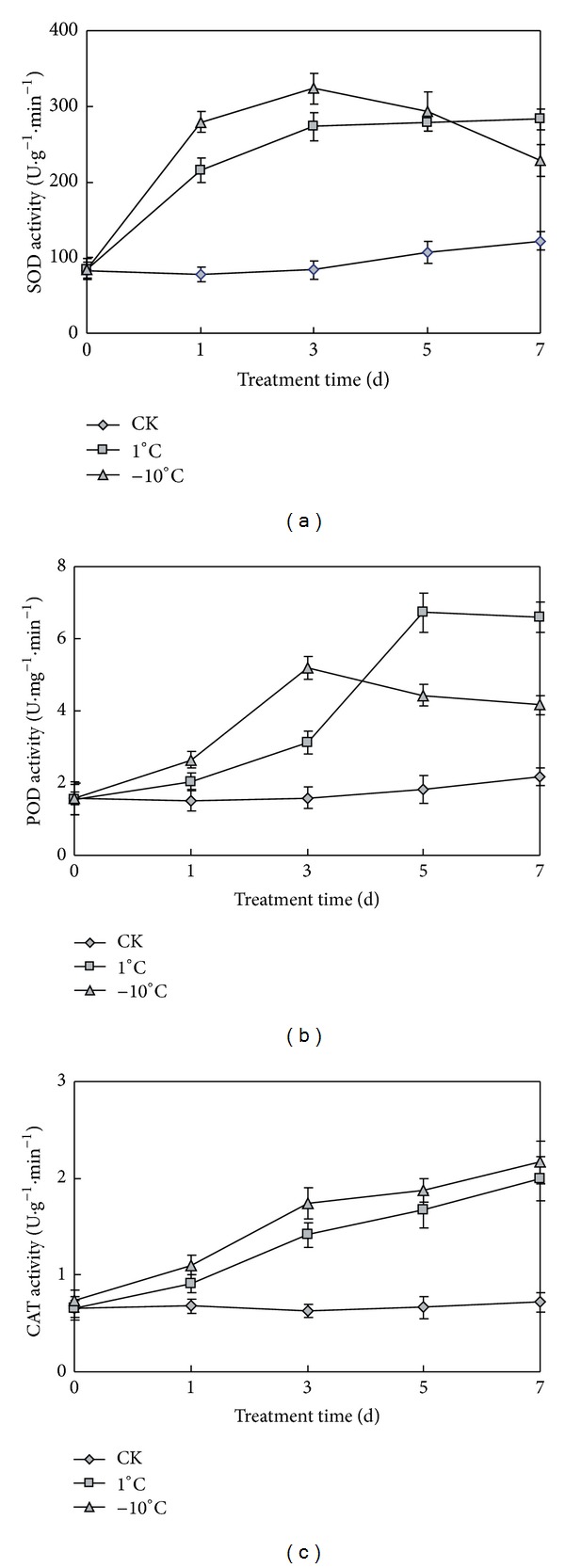
Activities of SOD (a), POD (b), and CAT (c) of seedling leaves under normal and low temperatures. CK represents normal growth condition. Each value is the average ± standard error (±SE) result of three independent measurements.

We measured the activities of these enzymes under normal and cold stress. Compared with normal, the SOD activity was higher under the cold treatment, as shown in Figure 6(a). At 1°C, the SOD activity was increased rapidly in the first three days and remained unchanged in later days. At −10°C, the SOD activity was increased first and decreased later. In the third day, the SOD activity was increased to the max value 323 U*·*g^−1^ *·*min^−1^, which was nearly 4 times of normal temperature. Then, the SOD activity was decreased rapidly to a low value of 229 U*·*g^−1^ *·*min^−1^ in the seventh day. At this time, the seedlings were damaged seriously by low temperature. Compared with normal, the SOD activity was higher than normal under the cold treatment. Increased SOD activity contributed for cold tolerance of naked oats under cold stress. Especially in the first day, the SOD activities had increased greatly, indicated that naked oats is resisting and adapting to low temperature, and contributed to reduce the damage by cold. The SOD activity decreased later at −10°C, which may be due to the greater damage by temperature of −10°C affected the synthesis of SOD in plant. 

POD activities were higher under low temperature than normal temperature as shown in Figure 6(b). At 1°C, POD activity was slowly increased in the first three days, rapidly increased in the third to fifth days, and reached the max of 6.7 U*·*min^−1^ *·*mg^−1^ in the fifth day, more than 4 times of control. Then POD activity slightly decreased to 6.6 U*·*mg^−1^ *·*min^−1^ in the 7th day, which was almost nearly the max value. At −10°C, POD activity was rapidly increased in the first three days and reached the max of 5.2 U*·*mg^−1^ *·*min^−1^ in the third day, more than 3 times of control. Then POD activity decreased in the third to seventh days. The POD activity decreased to 4.16 U*·*mg^−1^ *·*min^−1^ in the 7th day, which was still 2.5 times more than that of control. PODs are a large family of enzymes that typically catalyze peroxide. The increased POD activities under low temperature had improved cold tolerance in some degree. POD activities decreased greatly in later days, indicating that low temperature had affected POD enzyme. It maybe due to low temperature affected RNA transcription and translation, reducing the synthesis of POD. At the same time proline content also increased under low temperature, which can degrade peroxidase. It can also decrease the POD activity. 

Compared with normal, the CAT activity was higher under the cold treatment than normal temperature as shown in Figure 6(c). At 1°C, the CAT activity was increased with the prolongation of low temperature stress. CAT activity was 0.66 U*·*g^−1^ *·*min^−1^ before of cold treatment and increased to 1.99 U*·*g^−1^ *·*min^−1^ after the seven days cold treatment. At −10°C, the CAT activity was increased rapidly in the first three days and increased slowly in later days. In the third day, the CAT activity was increased to the 1.74 U*·*g^−1^ *·*min^−1^, which was nearly 2.5 times of normal temperature. Then, the CAT activity was increased to 2.16 U*·*g^−1^ *·*min^−1^ in the seventh day. Compared with normal, the CAT activity was higher than normal under the cold treatment. Increased CAT activity contributed to cold tolerance of naked oats under cold stress. 

In this experiment, the activities of these enzymes were increased in different degrees under cold stress. The results imply that higher SOD, POD, and CAT activities enhanced the capacity for scavenging ROS and contributed to enhanced tolerance of plant to cold stress. The result was similar to the SOD changes in other cold treatment plants.

## 4. Conclusions

*Avena nuda* L. is an important crop and has tolerance to low temperature. In the long time of evolution, it should have some cold resistant mechanism. The cold-stress leads to complex cellular and biochemical changes such as altered membrane composition and accumulation of proline, as well as the activities of antioxidant enzymes. In the present study, we have, for the first time, investigated the physiological changes in naked oats under low temperature. As results showed, low temperature stress caused a certain degree of physiological impairment in naked oats leaves. Here, we showed that cold increased the content of proline, MDA, electrolyte leakage, SOD, CAT, and POD activities. The content of chlorophyll was decreased. The study provided theoretical basis for cultivation and antibiotic breeding in *Avena nuda* L.
